# Refusal of PEG Feeding Following a Carotid Endarterectomy

**DOI:** 10.7759/cureus.3046

**Published:** 2018-07-25

**Authors:** Adam L Jones

**Affiliations:** 1 MEDIC, Cardiff University, Cardiff, GBR

**Keywords:** dysphagia, carotid endarterectomy, acute stroke, nasogastric tube feeding, autonomy, beneficence, right to refuse treatment, glossopharyngeal nerve injury

## Abstract

This case study presents a patient who has undergone right carotid endarterectomy complicated by glossopharyngeal nerve (or cranial nerve (CN) IX) injury. The patient had one transient ischaemic attack (TIA) three weeks before admission. A computed tomography (CT) scan two days after admission illustrated a right-sided parietal infarct. The patient subsequently had a CT angiogram, which showed a large, calcified plaque in the right internal carotid artery. He then underwent a right carotid endarterectomy. After the procedure, he developed dysphagia. A discussion was had with the patient about using percutaneous endoscopic gastrostomy (PEG) to provide a means of feeding. The patient subsequently refused this in favor of nasogastric tube (NGT) feeding despite the doctor’s advice. This highlights an important learning point with regards to patient autonomy and their right to refuse treatment. Further research is required into the quality of life after PEG to help patients make an informed decision.

## Introduction

Carotid endarterectomy is a surgical procedure in which atherosclerotic plaque is removed from a carotid artery to reduce the risk of stroke. The complications associated with this procedure range from hemorrhage, myocardial infarction, cranial nerve damage, stroke, and death [1-2].

Our patient (patient X) suffered a cranial nerve (CN) IX injury as a result of a right carotid endarterectomy. Research suggests this complication is uncommon (33/6,878, 0.5%) [3]. As a result, he then developed dysphagia (which is an expected outcome of a CN IX injury). A discussion followed with the patient about his best interests and using a percutaneous endoscopic gastrostomy (PEG) feed. The patient declined this and requested nasogastric tube (NGT) feeding instead. It is important to ensure that patient X is making an informed decision. Additionally, practitioners should consider the General Medical Council’s (GMC) guidelines and respect patient X’s autonomy because he is deemed to have capacity, even though he refuses interventions against clinical opinion [4].

## Case presentation

Patient X is a 78-year-old, right-handed man who was seen at the emergency department, presenting with sudden-onset left hemiparesis persisting for five hours. Prior to this, he complained of loss of vision temporarily in his right eye, which he described as a “film coming over his eye.” Swallowing and speech remained intact. Patient X’s medical history consisted of a transient ischaemic attack (TIA), which occurred three weeks ago, hypertension, ischaemic heart disease (coronary artery bypass surgery in 2008), and chronic kidney disease secondary to obstructive uropathy. His social issues revealed that he is also a smoker with a 31-pack-a-year history and drinks one pint a week. His family history showed that his father died at the age of 46 from a myocardial infarction. Patient X lives alone but has a partner who is able to provide support. He is retired but previously worked loading and driving lorries. Prior to the presentation, he was independent and able to carry out all his activities of daily living.

A general examination revealed a carotid bruit and a blood pressure of 151/60 mmHg. On neurological examination, he was alert and orientated. Left-sided power was reduced to 2/5 in the upper limb and 4/5 in the lower limb. There were a mild left-sided facial droop and a left-sided homonymous hemianopia. Reflexes were difficult to elicit. Sensation and coordination were intact.

Investigations

**Figure 1:**
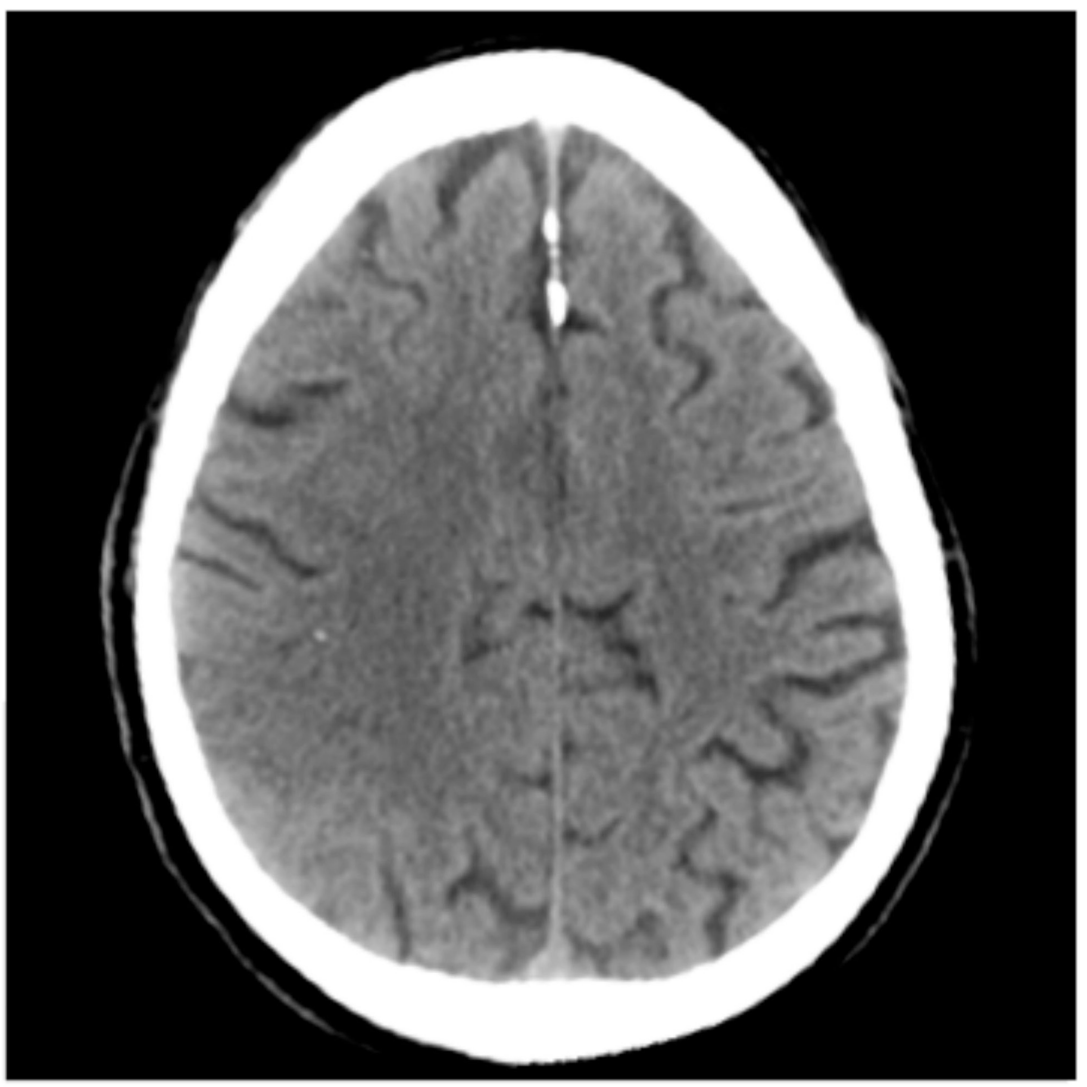
CT scan of the brain. Scan taken on admission reported as unremarkable by the consultant radiologist. CT: computed tomography

**Figure 2:**
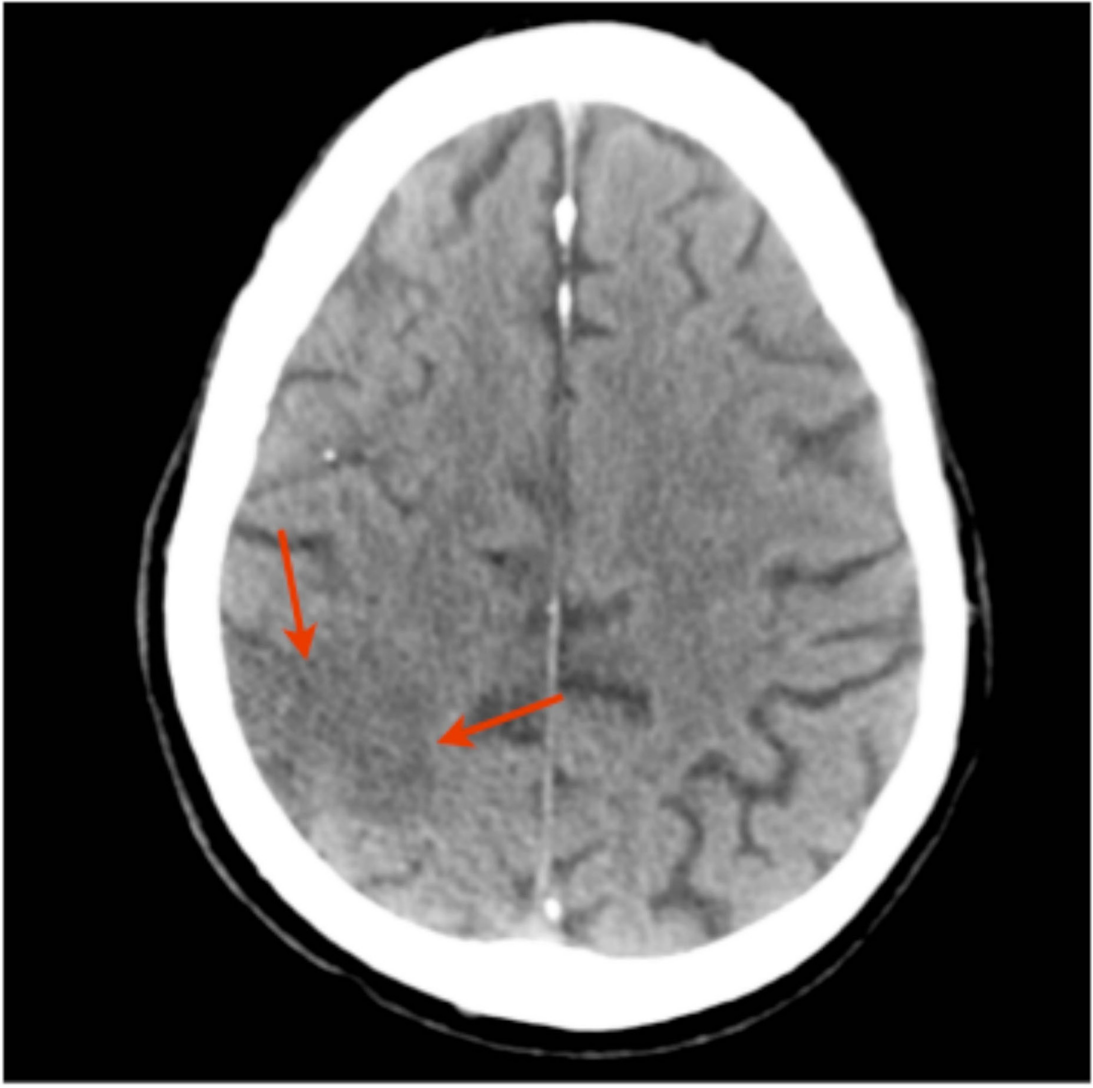
CT scan of the brain. Scan taken 48 hours post-admission. The report stated a right-sided hypodense area that was most likely to be a right-sided parietal infarct. CT: computed tomography

Initially, a computed tomography (CT) scan of the head was noted to be unremarkable (Figure 1) but a further scan (two days later) depicted a right-sided hypodense area, likely a right-sided parietal infarct (Figure 2).

The electrocardiogram (ECG) showed a sinus rhythm. A pattern of left ventricular hypertrophy was noted.

**Figure 3:**
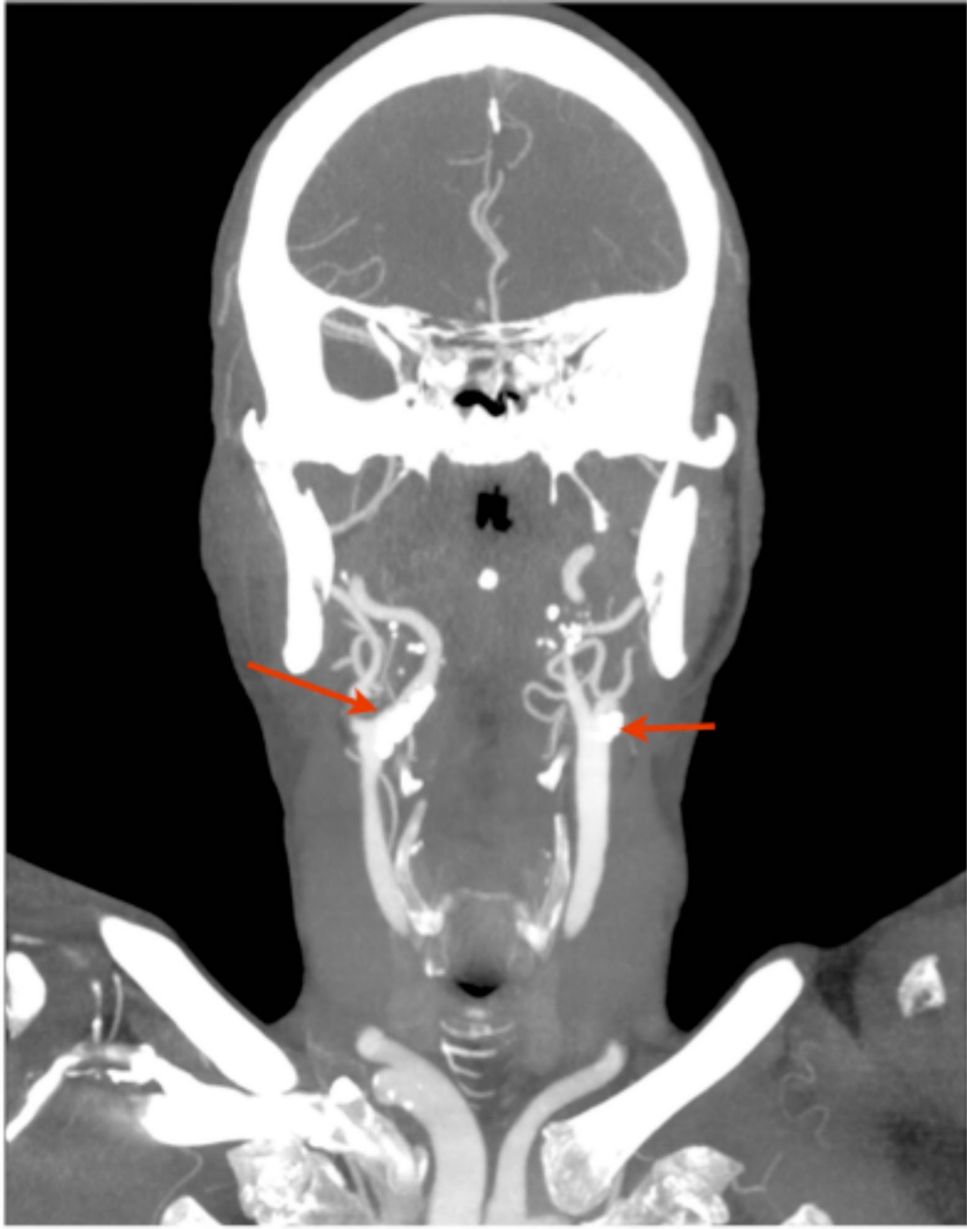
CT angiogram. Scan taken 10 days post-admission. The scan shows calcified plaques involving both carotid bifurcations larger on the right than the left. CT: computed tomography

A carotid scan, five days post-presentation showed a large calcified plaque at the origin of the right internal carotid artery. A subsequent CT angiogram, 10 days post-presentation demonstrated calcified plaques in both carotid bifurcations, which appeared larger in the right internal carotid artery compared to the left  (Figure 3).

Treatment 

Patient X didn’t present within the time window for thrombolysis, therefore, he was started on aspirin 300 mg daily for two weeks to reduce the risk of stroke [1]. The patient was transferred to an inpatient rehabilitation unit. Due to the scan findings, the patient underwent a right carotid endarterectomy (12 days post-admission). This procedure also aimed to reduce the risk of stroke [2].

Outcomes and follow-up

Two days following the right carotid endarterectomy, the patient developed dysphagia. A swallow test was conducted, which showed food getting stuck in the esophagus and then being regurgitated. A discussion followed with the patient about using PEG for feeding. It was explained that the dysphagia was a result of irreversible nerve (CN IX) damage secondary to the carotid endarterectomy. The patient was deemed to have capacity and was thus posed the option of choosing PEG (long-term) or NGT (short-term). Patient X refused PEG and instead opted for feeding via an NGT. Patient X also started receiving physiotherapy in an attempt to strengthen his throat muscles. Doctors are doubtful this patient will be able to regain the ability to swallow normally.

**Figure 4:**
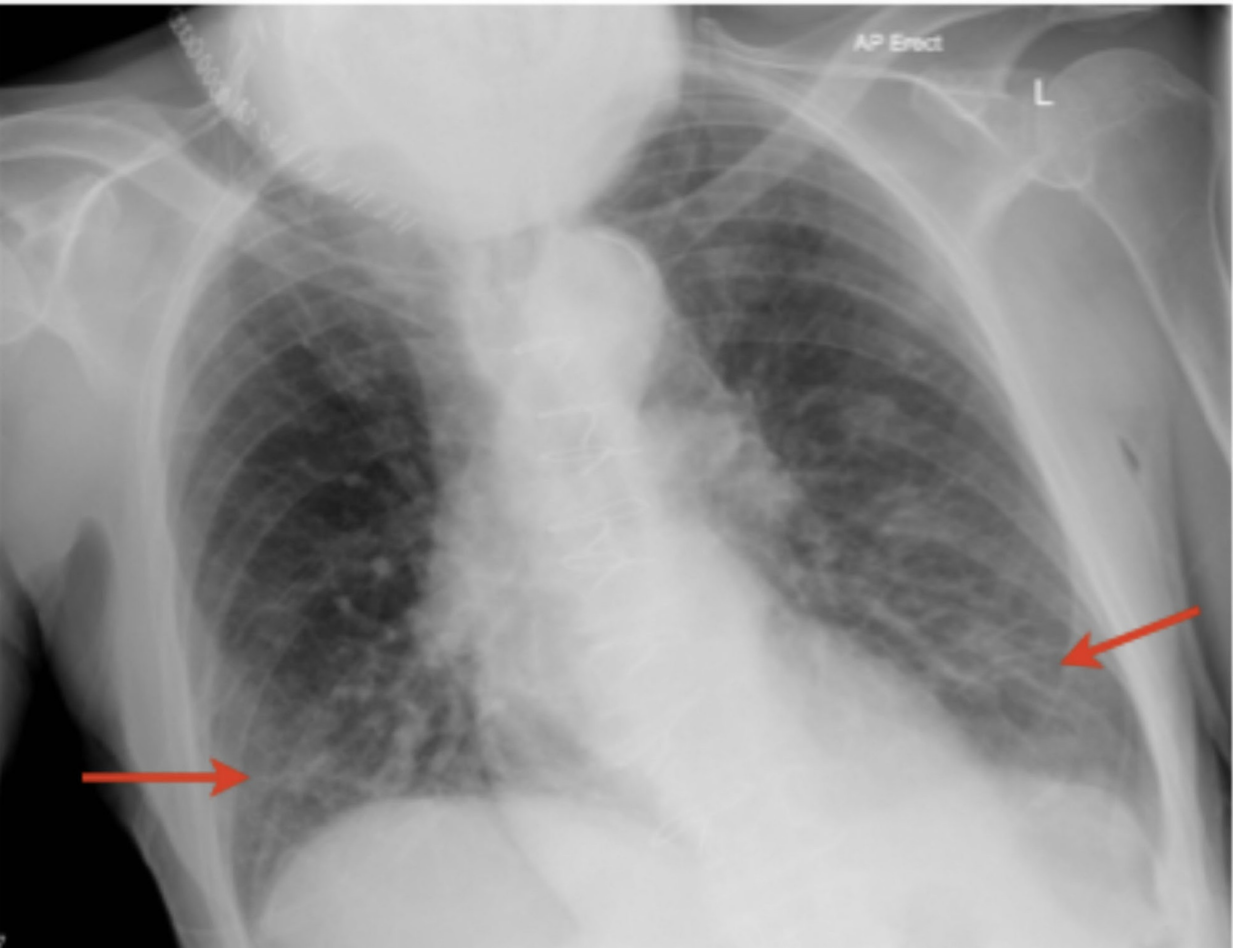
X-ray of chest. Scan taken 16 days post-admission, reported as having some minor airspace shadowing in both lower zones, which may be due to an infection.

Four days following the surgery, patient X developed healthcare-acquired pneumonia, confirmed by an X-ray (Figure 4). This was managed by intravenous antibiotics and chest physiotherapy.

Subsequently, patient X has recovered from pneumonia although his dysphagia is still present (one month postoperatively). He is currently admitted to the stroke unit for further rehabilitation.

## Discussion

NGT feeds are designed for short-term use (<4 weeks) [5]. One study by Gomes et al. found that NGT feeding had a higher probability of failure in comparison to PEG feeding [5]. Dwolatzky et al. found that the survival of patients with PEG was significantly higher than those with NGT [6]. Additionally, patients with PEG reported significantly fewer complications in comparison to those with NGT [6]. Patients with prolonged NGT may experience aspiration pneumonia, lesions of the nasal wing, chronic sinusitis, or gastro-oesophageal reflux [5,7-8]. For these reasons, the preferred method of providing enteral nutrition for an extended period of time is via a PEG.

Patient X required prolonged enteral nutrition, therefore, the doctor explained and recommended PEG feeding. PEG feeding after a stroke has been shown to improve nutritional status and reduce mortality [9-10]. However, this management option was refused by patient X because he believed having a PEG would be "admitting defeat" and explained that he found it difficult to accept the diagnosis at present. Patient X is competent and, therefore, has the autonomy to make informed treatment decisions; consequently, the doctor must respect this [11]. Deciding whether to feed via PEG is often very distressing for patients and their families, resulting in long-term implications [12].

The complex moral and ethical issues regarding this ethical dilemma result in a struggle between patient autonomy and the principle of beneficence and non-maleficence [13]. Patient autonomy allows patient X to make his own decisions and is a fundamental basis for clinical ethics [14]. Beneficence and non-maleficence refer to actions that benefit patient X and cause no harm [14]. This is difficult to achieve in this patient who refuses the PEG feed against medical advice, therefore, increasing the risk to himself [5,7-8]. Further support through specialist nurses, more time to make a decision, and an opportunity to talk to another patient who has a PEG may be beneficial in helping a patient make a well-informed decision regarding PEG feeding [12-14].

Patient X refused the PEG feeding tube because he believes that having it is “admitting defeat” and believes that the change to his body will portray him as “weak.” Similarly, Lin et al. concluded that one of the most common reasons for refusals of long-term PEG was "to keep the subjects’ body integrity" [15]. The psychological effects of having PEG must not be overlooked. These patients are at an increased risk of depression and stress due to major lifestyle changes [16]. These changes also affect the relatives of the patients, leading to high levels of stress due to their new-found roles as carers [16]. The importance of involving psychological and psychiatric services in these cases should be considered [16].

## Conclusions

This article illustrates the complex moral and ethical issues surrounding percutaneous endoscopic gastrostomy, highlighting the importance of providing patients with accurate information about the pros and cons of the proposed intervention, in order for them to make a fully informed decision. Although procedures may provide benefits to a patient, they may cause difficulties in the psychosocial aspects of their life. Therefore, all patients should be assessed individually. The benefits, risks, and subsequent implications should all be considered when deciding whether a patient should have a PEG tube placed. Further research is required into the quality of life after PEG to help patients make an informed decision.
